# Dietary Zinc Supplementation Prevents Autism Related Behaviors and Striatal Synaptic Dysfunction in *Shank3* Exon 13–16 Mutant Mice

**DOI:** 10.3389/fncel.2018.00374

**Published:** 2018-10-22

**Authors:** Chantelle Fourie, Yukti Vyas, Kevin Lee, Yewon Jung, Craig C. Garner, Johanna M. Montgomery

**Affiliations:** ^1^Department of Physiology and Centre for Brain Research, University of Auckland, Auckland, New Zealand; ^2^German Center for Neurodegenerative Disorders, Charité-Universitätsmedizin Berlin, Berlin, Germany

**Keywords:** autism, SHANK3, zinc, synapse, NMDA receptor

## Abstract

The SHANK family of synaptic proteins (SHANK1–3) are master regulators of the organizational structure of excitatory synapses in the brain. Mutations in *SHANK1–3* are prevalent in patients with autism spectrum disorders (ASD), and loss of one copy of *SHANK3* causes Phelan-McDermid Syndrome, a syndrome in which Autism occurs in >80% of cases. The synaptic stability of SHANK3 is highly regulated by zinc, driving the formation of postsynaptic protein complexes and increases in excitatory synaptic strength. As ASD-associated SHANK3 mutations retain responsiveness to zinc, here we investigated how increasing levels of dietary zinc could alter behavioral and synaptic deficits that occur with ASD. We performed behavioral testing together with cortico-striatal slice electrophysiology on a *Shank3*^−/−^ mouse model of ASD (*Shank3*^ex13–1616−/−^), which displays ASD-related behaviors and structural and functional deficits at striatal synapses. We observed that 6 weeks of dietary zinc supplementation in *Shank3*^ex13–16−/−^ mice prevented ASD-related repetitive and anxiety behaviors and deficits in social novelty recognition. Dietary zinc supplementation also increased the recruitment of zinc sensitive SHANK2 to synapses, reduced synaptic transmission specifically through *N*-methyl-D-aspartate (NMDA)-type glutamate receptors, reversed the slowed decay tau of NMDA receptor (NMDAR)-mediated currents and occluded long term potentiation (LTP) at cortico-striatal synapses. These data suggest that alterations in NMDAR function underlie the lack of NMDAR-dependent cortico-striatal LTP and contribute to the reversal of ASD-related behaviors such as compulsive grooming. Our data reveal that dietary zinc alters neurological function from synapses to behavior, and identifies dietary zinc as a potential therapeutic agent in ASD.

## Introduction

The SHANK family of proteins (SHANK1–3) are localized at the core of the postsynaptic density (PSD) at glutamatergic synapses where they bind to structural proteins, glutamate receptors, and the actin cytoskeleton to modulate the structure, plasticity and maturation of excitatory synapses (Boeckers et al., 2002; Kreienkamp, 2008). SHANK proteins display a temporal expression pattern at synapses, with SHANK2 being one of the first proteins concentrated at the developing PSD, followed by SHANK3 and SHANK1 (Bresler et al., 2004; Grabrucker et al., 2011a). SHANK3 strengthens glutamatergic α-amino-3-hydroxy-5-methyl-4-isoxazolepropionic acid receptor (AMPAR) and *N*-methyl-D-aspartate receptor (NMDAR) mediated synaptic transmission and increases glutamate release via the formation of trans-synaptic signaling complexes with neurexin and neuroligin (Arons et al., 2012). Multiple point mutations, deletions, and truncations occur in *SHANK1–3* in people with autism spectrum disorders (ASD), with *SHANK3* mutations mainly found in ASD individuals with moderate to severe intellectual disability (Leblond et al., 2014). In addition, loss of one copy of *SHANK3* causes Phelan-McDermid or 22q13 deletion syndrome, a syndrome in which Autism occurs in >80% of cases (Wilson et al., 2003; Phelan and McDermid, 2012; Soorya et al., 2013). ASD and 22q13 mutations in *SHANK3* disrupt AMPAR and NMDAR signaling and interfere with the ability of SHANK3 to alter presynaptic function via trans-synaptic signaling (Arons et al., 2012). Mouse models expressing ASD mutations in *Shank1–3* exhibit impaired glutamatergic synaptic function, decreased synaptic plasticity, increased anxiety and repetitive behaviors and impaired social interactions (Hung et al., 2008; Bozdagi et al., 2010; Peça et al., 2011; Schmeisser et al., 2012; Duffney et al., 2013; Lee et al., 2015; Speed et al., 2015; Mei et al., 2016). Moreover, restoring excitatory synaptic function (e.g., by altering NMDA or metabotropic-type glutamate receptor function, or restoring *Shank3* expression) improves ASD-related behaviors (Won et al., 2012; Chung et al., 2015; Lee et al., 2015; Mei et al., 2016). Therefore, glutamatergic synapses are a major focus for developing treatments for the behavioral deficits associated with ASD.

The C-terminal sterile alpha motif (SAM) domains in SHANK2 and SHANK3 are high affinity zinc binding sites (Boeckers et al., 2005), and zinc stabilizes synaptic SHANK localization and promotes the recruitment of pre- and postsynaptic protein complexes to excitatory synapses (Baron et al., 2006; Grabrucker et al., 2014; Arons et al., 2016). SHANK proteins containing ASD mutations retain their responsiveness to zinc: acute increases in zinc strengthen glutamatergic synaptic transmission and recruit postsynaptic protein complexes at excitatory synapses expressing ASD mutations in SHANK2 and SHANK3 (Lee et al., 2015; Arons et al., 2016), and trans-synaptic zinc mobilization reverses ASD-related behavioral deficits in *Shank2*^−/−^ mice (Lee et al., 2015). Zinc is stored in glutamatergic synaptic vesicles where it is co-released with glutamate (Assaf and Chung, 1984; Howell et al., 1984; Westbrook and Mayer, 1987; Mayer and Vyklicky, 1989; Smart et al., 1994). Zinc enters the postsynapse via ion channels where it modulates synaptic transmission and plasticity (Mayer and Vyklicky, 1989; Izumi et al., 2006; Takeda et al., 2010; Pan et al., 2011). Zinc depletion induces disintegration of SHANK3 complexes and weakens glutamatergic synaptic strength (Arons et al., 2016). In human studies, chronic zinc deficiency is a risk factor for ASD, and low zinc levels have been reported in children with ASD (Faber et al., 2009; Yasuda et al., 2011). Together these data suggest that zinc mismanagement is a major factor in regulating both SHANK proteins at the cellular level and ASD at the behavioral level.

Here, we sought to determine whether dietary zinc supplementation is a viable strategy to rescue synaptic and behavioral deficits in *Shank3*^ex13–16−/−^ mice that exhibit ASD-associated behavioral deficits and weakened synaptic function in the dorsolateral striatum (Peça et al., 2011). We performed behavioral and electrophysiological analysis on *Shank3*^ex13–16−/−^ mice fed normal (30 ppm) or supplemented (150 ppm) zinc levels (Tallman and Taylor, 2003; Grabrucker et al., 2011b). Our data show that dietary zinc supplementation prevented ASD-associated behaviors by altering synaptic transmission through NMDARs as well as long term plasticity at cortico-striatal synapses.

## Materials and Methods

### Animals

All animal experiments have been performed subject to regulations approved by the University of Auckland Animal Ethics Committee and adherence to the ARRIVE guidelines. *Shank3*^ex13–16−/−^ mice (B6.129-*Shank3*^tm2Gfng^/J; Peça et al., 2011) were imported from Jackson Laboratories, Bar Harbor, ME, USA and maintained at the University of Auckland, Auckland, New Zealand. Wild-type (WT), heterozygous and homozygous (Hom) mice were generated from heterozygous × heterozygous breeding pairs. All experimental animals were housed in a normal 12/12-h light dark cycle in groups of two to four per cage with mixed genotypes. Food and water were available *ad libitum*.

### Experimental Design

Animals were randomly assigned to a normal zinc diet (30 ppm) or a zinc supplementation diet (150 ppm; Tallman and Taylor, 2003; Tran et al., 2009; Grabrucker et al., 2011b), purchased from Research Diets, Brunswick, NJ, USA (catalogs D19410B and D06041101, respectively) for 6 weeks from weaning (postnatal day 21). Aside from the zinc levels, the two diets were identical egg white based rodent diets. No adverse effects on animal health or development were evident on either diet. Behavioral, electrophysiological, and imaging experiments were performed at 9–10 weeks of age on males and females: in total 69 WT (38 male, 31 female) and 68 Hom (37 male and 31 female). Genotypes were determined by PCR as previously described (Peça et al., 2011). All experiments and analyses were performed with the experimenter blinded to genotype and zinc diet by independent animal coding with a unique identification number at weaning.

### Behavioral Tests and Analysis

All behavioral experiments were recorded and analyzed with Ethovision (Noldus) software.

#### Grooming Behavior

Grooming behavior was recorded under low light conditions (40 lx), for 30 min and the time spent grooming during this period was timed. Grooming included face-wiping, scratching/rubbing of head and ears, grooming of tail and full-body grooming (Chung et al., 2015; Pearson et al., 2011).

#### Dark-Light Emergence Test

Testing was conducted in a two-chambered apparatus where one chamber was in complete darkness and the other light (508 lx), with a door allowing mice to freely move between the two chambers. Mice were placed in the dark chamber for 5 min before the door was opened and the test mouse allowed to freely explore the apparatus for 10 min. The percentage of time spent in the light vs. dark chambers was used as an index of anxiety-like behaviors (Peça et al., 2011).

#### Three-Chamber Social Interaction Assay

The three-chamber social interaction assay was conducted under low light conditions (40 lx) and consisted of three phases, as previously described (Won et al., 2012; Lee et al., 2015). In the first phase, the test mouse was placed in the center chamber of the apparatus with two small containers in the left and right chamber, and was allowed to explore the environment freely for 10 min for habituation. The mouse was then guided to the center chamber, and the two side entrances were blocked while a stranger mouse (Stranger 1) was placed in one of the containers. Then, the two entrances were opened to allow the mouse to explore the new environment freely for 10 min. In the third phase, the test mouse was guided to the center chamber, and the entrances to the side chambers closed. A novel stranger mouse (Stranger 2) was then introduced into the empty container while the familiar (Stranger 1) mouse remained in the other container. The entrances were opened and the test mouse was allowed to explore Stranger 1 and 2 for 10 min. Social interaction was defined as the time the test mouse spent sniffing, orienting its nose towards, or interacting with the stranger mouse.

### Cortico-Striatal Electrophysiology

#### Slice Preparation

Acute coronal cortico-striatal brain slices were prepared from 9 to 10 week-old mice, based on previous studies (Peça et al., 2011; Ting et al., 2014). Mice utilized for electrophysiological analysis were independent from mice used for behavioral analysis. Mice were culled with CO_2_, decapitated and the brains removed into carbogenated (95% O_2_, 5% CO_2_) ice-cold protective cutting artificial cerebrospinal fluid (ACSF) with the composition (in mM): 93 NMDG, 2.5 KCl, 1.25 NaH_2_PO_4_, 30 NaHCO_3_, 20 HEPES, 25 glucose, 2 thiourea, 5 L-ascorbic acid, 3 Na-pyruvate, 0.5 CaCl_2_, 10 MgSO_4_.7H_2_O, (pH 7.4, osmolarity of 295–305 mOsm). The brains were rapidly sectioned at 300 μm using a vibratome and slices transferred for recovery in protective cutting ACSF at 34°C. Slices were maintained at room temperature (RT) in a holding chamber in carbogenated recording ACSF with the composition (in mM): 97 NaCl, 2.5 KCl, 1.25 NaH_2_PO_4_, 30 NaHCO_3_, 25 glucose, 20 HEPES, 2 CaCl_2_, 2 MgSO_4_.7H_2_O, 2 thiourea, 5 L-ascorbic acid, 3 Na-pyruvate (pH 7.4 with osmolarity of 295–305 mOsm). Fast inhibitory currents were not blocked. The dorsolateral striatum and individual medium spiny neurons (MSNs) were visualized with a Zeiss Axioskop microscope equipped with IR-DIC optics.

#### Presynaptic Stimulation and Postsynaptic Current Recording

A platinum iridium concentric bipolar stimulating electrode was placed on the inner border of the corpus callosum between the cortex and dorsolateral striatum for presynaptic stimulation of glutamatergic inputs to the dorsolateral striatum. Stimulation was performed with a Digitimer constant current stimulator with pulses delivered at 0.1 Hz. Whole-cell patch-clamp recordings were obtained from MSNs using glass recording electrodes (resistance 6–8 MΩ) filled with internal solution [in mM: 120 K gluconate (or 120 Cs gluconate for NMDAR EPSCs), 40 HEPES, 5 MgCl_2_, 2 NaATP, 0.3 NaGTP and 5 QX314 (for NMDAR EPSCs), pH 7.2, 298mOsm]. Membrane currents and potentials were processed with a Multiclamp 700B commander (Axon Instruments, California, CA, USA) and digitized at 10 KHz (Digidata 1440, Axon Instruments, California, CA, USA) to convert analog to digital signals. Events were sampled at 10 KHz and low-pass filtered at 1 KHz. Series resistance (Rs) was measured and recordings with Rs variation greater than 20% were discarded. All data acquisition and analysis were performed using pClamp 10 acquisition software and Clampfit 10, respectively (Axon Instruments, California, CA, USA).

#### AMPA- and NMDA Receptor Current Recording and Analysis

The maximum AMPAR mediated excitatory postsynaptic current (EPSC) amplitude was determined and the stimulator set to deliver pulses that produced 50% of this amplitude. To record isolated NMDAR EPSCs, 10 μM 6-Cyano-7-nitroquinoxaline-2,3-dione (CNQX) was bath applied to block the AMPAR EPSCs and each neuron voltage clamped at +40 mV. NMDAR EPSCs were measured in response to presynaptic stimulation (50 μA pulses at 0.1 Hz stimulation). NMDAR decay kinetics were measured as previously described (Cathala et al., 2000): a double exponential function was fitted from the current peak to the baseline: *I*(t) = *I*_f_exp((−t/τ_f_) + *I*_s_exp((−t/τ_s_), where *I*_f_ and *I*_s_ are the amplitudes of the fast and slow decay components, and τ_f_ and τ_s_ are their respective decay time constants. To compare decay times between different experiments we used a weighted mean decay time constant: τ_w_ = [*I*_f_/(*I*_f_ + *I*_s_)]/τ_f_ +[*I*_s_/(*I*_f_ + *I*_s_)]/τ_s_.

#### Synaptic Plasticity

For long term potentiation (LTP) experiments, a 5-min baseline of AMPAR EPSCs was recorded in response to 0.1 Hz presynaptic stimulation. LTP was then induced via four presynaptic stimulation trains of 100 Hz for 1 s, with a 10 s interval between trains while the postsynaptic cell was voltage clamped at 0 mV (Fino et al., 2005). The post-LTP induction EPSCs were subsequently recorded at 0.1 Hz. The omission of extracellular Mg^2+^, nor the inclusion of GABA_A_ receptor blockers was required for the induction of LTP. In previous studies, high frequency stimulation has also been shown to induce long-term depression (LTD) of AMPAR EPSCs in MSNs in the striatum (Calabresi et al., 1993; Walsh, 1993; Lovinger, 2010). However, it should be noted that the placement of the stimulating electrode together with the level of postsynaptic depolarization play a major role in the LTP vs. LTD induction protocol (Spencer and Murphy, 2000).

### Immunohistochemistry, Confocal Imaging and Image Analysis

We performed immunohistochemistry on 50 μm coronal sections from 4% paraformaldehyde immersion fixed WT and *Shank3*^ex13–16−/−^ mice fed normal (30 ppm) and supplemented (150 ppm) zinc levels for 6 weeks. The sections were permeablized overnight with 0.25% Triton X-100 in 1 × PBS (PBST) at 4°C, non-specific binding was blocked by incubation in 10% normal goat serum in 1× PBST and then sections were immunostained for SHANK2 (1:500, Santa Cruz Biotechnology), VGluT1 (1:500, Neuromab) or VGluT2 (Synaptic Systems, 1:500) for 72 h at 4°C. The sections were washed in 1 × PBST, incubated for 4 h at RT with secondary antibodies, then washed and incubated with Hoechst (Sigma) for 20 min at RT and slide mounted. The dorsal striatum was imaged via high-resolution confocal microscopy (OLYMPUS FV1000) at 60× magnification (1.35 NA) with 3× digital zoom using FluoView 3.0 image acquisition software. Laser power, amplifier gain and offset were optimized for each antibody to accommodate the dynamic range of the signal, and then settings were kept consistent for all subsequent imaging. For each section, z-stacks were obtained (10 images taken 0.5 μm apart) from three regions within the dorsal striatum. Puncta-by-puncta analysis was performed with ImageJ software where images were thresholded to select puncta above image background, and then the maximum intensity of SHANK2 co-localized with VGluT1 or VGluT2 (synaptic SHANK2) and SHANK2 not co-localized with VGluT1 or VGluT2 (non-synaptic SHANK2) were measured within the same field of view. The 3D Objects Counter tool was utilized in ImageJ to analyze puncta and identify Shank2 and VGluT1/2 colocalization in a 3-dimensional space captured by the z-stack. Each plane of the z-stack was analyzed individually to ensure only SHANK2 and VGluT1/2 puncta truly co-localized in each z-plane were captured.

### Statistical Analysis

All data represent mean ± SEM. Statistical analyses were performed using Graphpad Prism 6.0, with a *p* value < 0.05 considered significant. Tests for normality and homogeneity of variances were performed with the Shapiro-Wilk normality test and the Browne-Forsythe and Bartlett’s tests for variance, to determine whether parametric vs. non-parametric testing was applied. Details of each statistical test for each data set (one-way ANOVA with *post hoc* comparison, Kruskal Wallis or two-tailed student’s *t*-test) are provided in the figure legends.

## Results

### ASD-Related Behavioral Deficits Can Be Reversed by Dietary Zinc Supplementation

**Figure 1:**
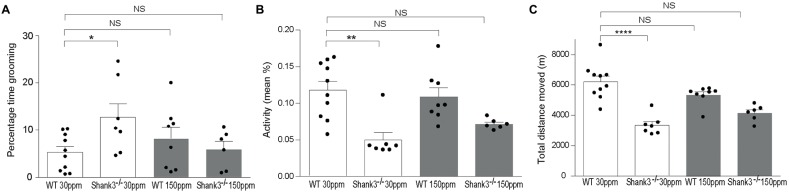
*Shank3*^ex13–16−/−^ autism spectrum disorder (ASD) mice repetitive grooming behaviors and reduced exploration behaviors are prevented with supplementation of dietary zinc. **(A)** Repetitive grooming in $Shank3_{30\text{ ppm}}^{\text{ex1}3-16-/-}$ mice was prevented in mice fed 150 ppm dietary zinc ($Shank3_{150\text{ ppm}}^{\text{ex1}3-16-/-}$). **(B)** Reduced exploratory behaviors in $Shank3_{30\text{ ppm}}^{\text{ex1}3-16-/-}$ mice, measured as a reduction in mean activity levels were no longer significantly different from Wild-type (WT) after dietary zinc supplementation. **(C)** Total distance moved by each mouse during the grooming test. Distance moved by *Shank3*^ex13–16−/−^ mice was increased with 150 ppm dietary zinc ($Shank3_{150\text{ ppm}}^{\text{ex1}3-16-/-}$). All data represent mean ± SEM. Statistics: one-way ANOVA with Dunnett’s *post hoc* test for **(A)** Kruskal-Wallis one-way ANOVA with Dunn’s *post hoc* test was applied for **(B,C)** as not all groups were normally distributed and variances were not equal (see “Materials and Methods” section). WT_30 ppm_ *n* = 10, $Shank3_{30\text{ ppm}}^{\text{ex1}3-16-/-}$ *n* = 7, WT_150 ppm_ *n* = 8, $Shank3_{150\text{ ppm}}^{\text{ex1}3-16-/-}$ *n* = 6 animals, *p < 0.05, **p < 0.01, ****p < 0.0001, NS, not significant.

To determine whether chronic changes in dietary zinc can alter the ASD-associated synaptic and behavioral deficits that occur in the striatum of the *Shank3*^ex13–16−/−^ ASD mouse model (Peça et al., 2011), *Shank3*^ex13–16−/−^ mice were fed normal (30 ppm) or high (150 ppm) dietary zinc levels for 6 weeks followed by analysis of ASD-related behaviors as well as glutamatergic excitatory synaptic transmission and plasticity in the cortico-striatal pathway. As expected, repetitive grooming behaviors in homozygous mice receiving normal dietary zinc ($Shank3_{30\text{ ppm}}^{\text{ex1}3-16-/-}$) were significantly increased compared to WT controls on the normal zinc diet (WT_30 ppm_) (Figure 1A; percentage time grooming WT_30 ppm_: 5.28 ± 1.23, $Shank3_{30\text{ ppm}}^{\text{ex1}3-16-/-}$ 12.70 ± 2.92, *p* < 0.05; Peça et al., 2011). Increasing dietary zinc in WT mice (WT_150 ppm_) resulted in no significant change in grooming behavior (percentage time grooming WT_150 ppm_ 8.24 ± 2.35; *p* > 0.05, Figure 1A). Strikingly, the repetitive grooming behavior observed in *Shank3*^ex13–16−/−^ mice was prevented when these mice were fed increased dietary zinc levels, such that the time spent grooming was no longer significantly different from WT mice (percentage time grooming $Shank3_{150\text{ ppm}}^{\text{ex1}3-16-/-}$ 6.00 ± 1.67, *p* > 0.05; Figure 1A). *Shank3*^ex13–16−/−^ mice also displayed significantly decreased mean activity levels and total distance moved within the grooming arena compared with WT mice (Figures 1B,C; mean activity: WT_30 ppm_ 0.118 ± 0.012%, $Shank3_{30\text{ ppm}}^{\text{ex1}3-16-/-}$ 0.05 ± 0.01%, *p* < 0.01; total distance moved WT_30 ppm_ 6206.53 ± 366.88 cm, $Shank3_{30\text{ ppm}}^{\text{ex1}3-16-/-}$ 3339.97 ± 241.51 cm, *p* < 0.01). With increased dietary zinc, mean activity levels and total distance moved were no longer significantly decreased in $Shank3_{150\text{ ppm}}^{\text{ex1}3-16-/-}$ mice compared to WT controls (mean activity: $Shank3_{150\text{ ppm}}^{\text{ex1}3-16-/-}$ 0.072 ± 0.003%, *p* > 0.05; total distance moved $Shank3_{150\text{ ppm}}^{\text{ex1}3-16-/-}$ 4143.97 ± 219.47 cm, *p* > 0.05; Figures 1B,C). High dietary zinc did not significantly alter the activity and movement of WT mice (mean activity: WT_150 ppm_ 0.109 ± 0.012%; total distance moved WT_150 ppm_ 5326.71 ± 216.02 cm, *p* > 0.05 compared with WT_30 ppm_ mice in both cases).

**Figure 2:**
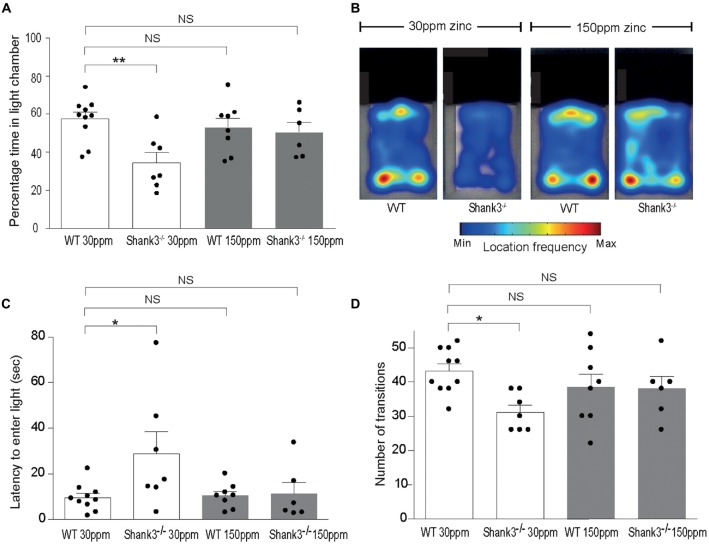
ASD anxiety behaviors in *Shank3*^ex13–16−/−^ mice are prevented with supplementation of dietary zinc. **(A)** Increased anxiety-type behaviors in $Shank3_{30\text{ ppm}}^{\text{ex1}3-16-/-}$ mice, reflected as a reduced percentage time spent in the light chamber, was prevented in mutant mice fed 150 ppm dietary zinc ($Shank3_{150\text{ ppm}}^{\text{ex1}3-16-/-}$). **(B)** Heat map example of WT and *Shank3*^ex13–16−/−^ mice on 30 or 150 ppm dietary zinc. Note the significantly decreased time the $Shank3_{30\text{ ppm}}^{\text{ex1}3-16-/-}$ mice spend in the light, and how this increases in $Shank3_{150\text{ ppm}}^{\text{ex1}3-16-/-}$ mice. **(C)** $Shank3_{30\text{ ppm}}^{\text{ex1}3-16-/-}$ mice take significantly longer to first exit the dark chamber (latency), reflecting heightened anxiety, and this was prevented in mutant mice fed 150 ppm dietary zinc ($Shank3_{150\text{ ppm}}^{\text{ex1}3-16-/-}$). **(D)** The number of transitions between the light and dark chambers are significantly reduced in $Shank3_{30\text{ ppm}}^{\text{ex1}3-16-/-}$ mice, but no longer significantly different from WT controls in *Shank3*^ex13–16−/−^ mice fed 150 ppm dietary zinc. All data represent mean ± SEM. Statistics: one-way ANOVA with Dunnett’s *post hoc* test. WT_30 ppm_ *n* = 10, $Shank3_{30\text{ ppm}}^{\text{ex1}3-16-/-}$ *n* = 7, WT_150 ppm_ *n* = 8, $Shank3_{150\text{ ppm}}^{\text{ex1}3-16-/-}$ *n* = 6 animals, *p < 0.05, **p < 0.01, NS, not significant.

$Shank3_{30\text{ ppm}}^{\text{ex1}3-16-/-}$ mice also displayed increased anxiety behavior, as assessed by the significant decrease in the percentage time spent in the light chamber in the dark-light emergence test compared with WT mice (WT_30 ppm_ 57.44 ± 3.54%, $Shank3_{30\text{ ppm}}^{\text{ex1}3-16-/-}$ 34.48 ± 5.41%; Figures 2A,B; *p* < 0.01; Peça et al., 2011). Increased dietary zinc did not significantly alter the percentage of time the WT mice spent in the light chamber (WT_150 ppm_ 53.25 ± 4.73%, *p* > 0.05; Figures 2A,B). *Shank3*^ex13–16−/−^ mice fed high dietary zinc however spent a similar time in the light chamber as WT control mice, showing that the increase in anxiety in *Shank3*^ex13–16−/−^ mice was also prevented by dietary zinc supplementation, rendering anxiety levels similar to control animals ($Shank3_{150\text{ ppm}}^{\text{ex1}3-16-/-}$ 50.50 ± 5.11%; Figures 2A,B). *Shank3*^ex13–16−/−^ mice on the normal zinc diet showed an increased latency to enter the light chamber compared with WT mice on the same zinc diet (Figure 2C; latency WT_30 ppm_ 9.59 ± 1.83 s, $Shank3_{30\text{ ppm}}^{\text{ex1}3-16-/-}$ 28.91 ± 9.56 s, *p* < 0.05), and this latency returned to control levels after the *Shank3*^ex13–16−/−^ mice were fed the high zinc diet (latency in $Shank3_{150\text{ ppm}}^{\text{ex1}3-16-/-}$ mice 11.29 ± 4.97 s; *p* > 0.05 compared to WT_30 ppm_; Figure 2C). WT mice fed the high zinc diet were not significantly different from WT mice on the control zinc diet (latency for WT_150 ppm_ mice 10.40 ± 1.93 s, *p* > 0.05 compared to WT_30 ppm_; Figure 2C). *Shank3*^ex13–16−/−^ mice on the normal zinc diet also transitioned between the light and dark chambers significantly less than WT mice on the same diet (Figure 2D; number of transitions: WT_30 ppm_ 43.20 ± 2.07, $Shank3_{30\text{ ppm}}^{\text{ex1}3-16-/-}$ 31.14 ± 2.09; *p* < 0.05), however with increased dietary zinc $Shank3_{150\text{ ppm}}^{\text{ex1}3-16-/-}$ mice displayed a similar number of transitions to WT mice ($Shank3_{150\text{ ppm}}^{\text{ex1}3-16-/-}$ 38.00 ± 3.58; *p* > 0.05). No significant effect was observed for WT mice fed the high zinc diet (WT_150 ppm_ 38.50 ± 3.83, *p* > 0.05; Figure 2D).

**Figure 3:**
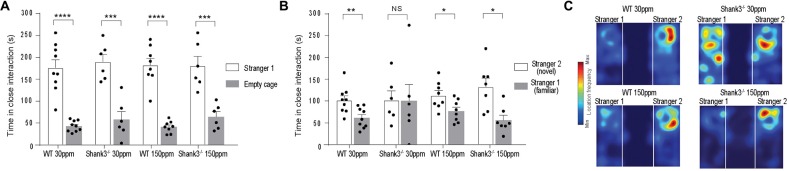
ASD social interaction deficits in *Shank3*^ex13–16−/−^ mice are prevented by increased dietary zinc. **(A)** All mice display normal social interaction during phase two of the three-chamber test. **(B)** $Shank3_{30\text{ ppm}}^{\text{ex1}3-16-/-}$ mice lack social novelty recognition during phase three, displaying no preference for the stranger or familiar mouse. Increased dietary zinc recovered the social novelty recognition behavior in *Shank3*^ex13–16−/−^ mice. **(C)** Example heat maps of phase three of the social interaction test. WT mice on the normal and high zinc diet prefer the novel mouse as depicted by increased presence close to stranger 2. *Shank3*^ex13–16−/−^ mice on the normal zinc diet show no preference for the novel mouse (stranger 2), but do show increased interaction with the novel mouse when fed the high zinc diet. All data represent mean ± SEM, two-tailed paired *t*-test. WT_30 ppm_ *n* = 9, $Shank3_{30\text{ ppm}}^{\text{ex1}3-16-/-}$ *n* = 6, WT_150 ppm_ *n* = 8, $Shank3_{150\text{ ppm}}^{\text{ex1}3-16-/-}$ *n* = 7 mice. *p < 0.05, **p < 0.01, ***p < 0.001, ****p < 0.0001, NS, not significant.

We also examined social interaction in WT and *Shank3*^ex13–16−/−^ mice fed with normal and supplemented zinc levels. WT and *Shank3*^ex13–16−/−^ mice on either the normal or high zinc diet displayed normal social interaction during the phase II of the three-chamber social interaction test (Figure 3A; time sniffing stranger 1 vs. empty cage respectively, *p* < 0.0001 in all cases: WT_30 ppm_ 176.00 ± 18.39 s vs. 42.78 ± 4.18 s; $Shank3_{30\text{ ppm}}^{\text{ex1}3-16-/-}$ 190.00 ± 16.98 s vs. 58.00 ± 18.08 s; WT_150 ppm_ 182.13 ± 15.78 s vs. 41.00 ± 4.72 s; $Shank3_{150\text{ ppm}}^{\text{ex1}3-16-/-}$ 180.50 ± 21.52 s vs. 64.33 ± 11.55 s). In phase three of the social interaction test, $Shank3_{30\text{ ppm}}^{\text{ex1}3-16-/-}$ mice lacked social novelty recognition, showing no significant preference for the novel mouse in contrast to WT mice (time in close interaction with stranger 2 (novel) vs. stranger 1 (familiar): WT_30 ppm_ 101.56 ± 10.27 s vs. 61.44 ± 7.86 s, *p* < 0.01; $Shank3_{30\text{ ppm}}^{\text{ex1}3-16-/-}$ 101.83 ± 21.22 s vs. 100.17 ± 38.08 s, *p* > 0.05; Figure 3B). Similar to the other ASD behaviors, dietary zinc supplementation in *Shank3*^ex13–16−/−^ mice also prevented the deficit in social novelty recognition as observed by the significant difference in time spent with the novel vs. familiar mouse in $Shank3_{150\text{ ppm}}^{\text{ex1}3-16-/-}$ mice (Figures 3B,C; WT_150 ppm_ 112.88 ± 11.01 s vs. 76.88 ± 8.87 s, *p* < 0.05; $Shank3_{150\text{ ppm}}^{\text{ex1}3-16-/-}$ 132.77 ± 19.86 s vs. 56.09 ± 11.23 s, *p* < 0.05).

### Dietary Zinc Levels Also Affect Synaptic Function and Plasticity in Shank3^ex13–16−/−^ Mice

**Figure 4:**
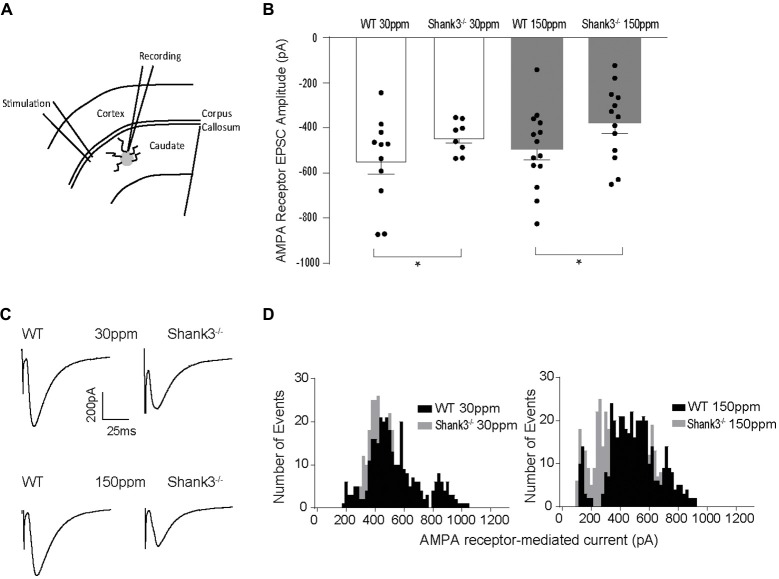
Effect of dietary zinc on cortico-striatal AMPAR mediated EPSCs in WT and *Shank3*^ex13–16−/−^ mice. **(A)** Schematic of electrode placement in dorsolateral striatum for all electrophysiology experiments. **(B)** *Shank3*^ex13–16−/−^ induces a decrease in AMPAR EPSC amplitude, and this decrease is not altered when animals are fed a high zinc diet (WT_30 ppm_ *n* = 11 cells from nine animals, $Shank3_{30\text{ ppm}}^{\text{ex1}3-16-/-}$ *n* = 8 cells from seven animals, WT_150 ppm_ *n* = 14 cells from nine animals, $Shank3_{150\text{ ppm}}^{\text{ex1}3-16-/-}$ *n* = 13 cells from eight animals). **(C)** Example AMPAR mediated EPSCs from each animal genotype and zinc diet. **(D)** Frequency histograms of AMPAR EPSC amplitudes from WT and *Shank3*^ex13–16−/−^ mice fed normal (left) or high zinc diet (right), illustrating the shift towards smaller AMPAR EPSC amplitudes in *Shank3*^ex13–16−/−^ mice on either zinc diet. All data represent mean ± SEM. *p < 0.05, student’s *t*-test.

Glutamatergic and GABAergic synapses are regulated by zinc, where it alters ion channel function (such as NMDA and GABA receptors), synaptic transmission, as well as the recruitment and stability of postsynaptic proteins including SHANKs (Mayer et al., 1989; Chen et al., 1997; Baron et al., 2006; Lee et al., 2015; Arons et al., 2016). As synaptic changes have been strongly linked to ASD-related behaviors (Won et al., 2012; Chung et al., 2015; Lee et al., 2015; Mei et al., 2016), we examined whether the observed dietary zinc-induced changes in ASD repetitive, anxiety, and social behaviors were accompanied by changes in function and plasticity at excitatory glutamatergic synapses in the dorsolateral striatum (Figures 4A–D). Cortico-striatal excitatory synaptic function was measured in acute slices prepared from WT and *Shank3*^ex13–16−/−^ mice fed either normal (30 ppm) or supplemented (150 ppm) dietary zinc. Evoked AMPAR-mediated EPSC amplitudes were found to be significantly decreased in $Shank3_{30\text{ ppm}}^{\text{ex1}3-16-/-}$ mice compared to WT_30 ppm_ mice (average EPSC amplitudes were WT_30 ppm_ −576.02 ± 56.09 pA, $Shank3_{30\text{ ppm}}^{\text{ex1}3-16-/-}$ −442.24 ± 25.66 pA, *p* < 0.05), as also illustrated by a significant leftward shift towards smaller AMPAR EPSC amplitudes and a higher frequency of smaller amplitude events (Figures 4B–D). Average evoked AMPAR mediated EPSC amplitudes remained unchanged with increased dietary zinc levels, showing that increasing dietary zinc levels did not significantly alter the difference in AMPAR-mediated synaptic transmission at cortico-striatal synapses between WT and *Shank3*^ex13–16−/−^ mice (average EPSC amplitudes were WT_150 ppm_ −536.37 ± 40.81 pA, $Shank3_{150\text{ ppm}}^{\text{ex1}3-16-/-}$ −378.56 ± 45.36 pA, *p* < 0.05; Figures 4B–D).

**Figure 5:**
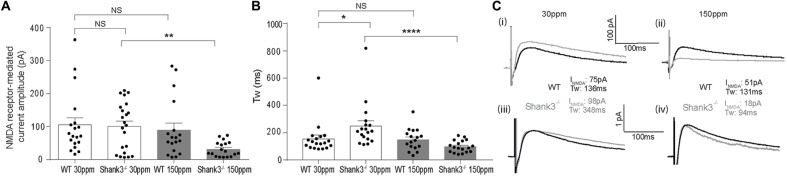
Increased dietary zinc specifically decreases cortico-striatal *N*-methyl-D-aspartate receptor (NMDA) receptor (NMDAR) mediated EPSCs in $Shank3_{150\text{ ppm}}^{\text{ex1}3-16-/-}$ mice. **(A)** *Shank3*^ex13–16−/−^ deletion ($Shank3_{30\text{ ppm}}^{\text{ex1}3-16-/-}$) had no effect on mean NMDAR mediated EPSC amplitude, but *Shank3*^ex13–16−/−^ mice fed 150 ppm dietary zinc ($Shank3_{150\text{ ppm}}^{\text{ex1}3-16-/-}$) exhibited a decreased mean NMDAR EPSC amplitude (Kruskal-Wallis one-way ANOVA with Dunn’s *post hoc* test; WT_30 ppm_ *n* = 19 cells from 10 animals, $Shank3_{30\text{ ppm}}^{\text{ex1}3-16-/-}$ *n* = 22 cells from nine animals, WT_150 ppm_ *n* = 18 cells from nine animals, $Shank3_{150\text{ ppm}}^{\text{ex1}3-16-/-}$ *n* = 18 cells from eight animals). **(B)** NMDAR decay kinetics, measured as the weighted time constant tau (τ_w_), were significantly slowed in $Shank3_{30\text{ ppm}}^{\text{ex1}3-16-/-}$ mice. Increased dietary zinc in *Shank3*^ex13–16−/−^ mice ($Shank3_{150\text{ ppm}}^{\text{ex1}3-16-/-}$) reversed the slowed kinetics (Kruskal-Wallis one-way ANOVA with Dunn’s *post hoc* test). **(C)** Example NMDAR EPSCs from both genotypes and dietary zinc levels. (i) NMDAR EPSCs from WT (black) and *Shank3*^ex13–16−/−^ (gray) mice on 30 ppm diet. (ii) NMDAR EPSCs from WT (black) and *Shank3*^ex13–16−/−^ (gray) mice on 150 ppm diet. (iii) Scaled NMDAR EPSCs from WT (black) and *Shank3*^ex13–16−/−^ (gray) mice on 30 ppm diet. (iv) Scaled NMDAR EPSCs from WT (black) and *Shank3*^ex13–16−/−^ (gray) mice on 150 ppm diet. All data represent mean ± SEM. *p < 0.05, **p < 0.01, ****p < 0.0001, NS, not significant.

In contrast to AMPAR-mediated currents, NMDAR-mediated EPSC amplitudes were not significantly different between WT and *Shank3*^ex13–16−/−^ mice fed normal dietary zinc (Figure 5A; average NMDAR EPSC amplitudes were: WT_30 ppm_ 105.03 ± 23.14 pA, $Shank3_{30\text{ ppm}}^{\text{ex1}3-16-/-}$ 100.87 ± 16.93 pA, *p* > 0.05). Increasing dietary zinc in WT animals also had no effect on NMDAR-EPSC amplitudes (Figure 5A; WT_150 ppm_ 89.55 ± 25.31 pA). However, dietary zinc supplementation in *Shank3*^ex13–16−/−^ mice induced a marked reduction in NMDAR-mediated EPSC amplitudes (Figure 5A), $Shank3_{150\text{ ppm}}^{\text{ex1}3-16-/-}$ 38.60 ± 13.78 pA, *p* < 0.05).

We also examined the decay kinetics of the evoked NMDAR mediated EPSCs in both WT and *Shank3*^ex13–16−/−^ mice fed with normal and high zinc. The decay kinetics of NMDAR-mediated EPSCs were significantly slowed in $Shank3_{30\text{ ppm}}^{\text{ex1}3-16-/-}$ mice compared to WT_30 ppm_ mice (Figure 5B; weighted tau: WT_30 ppm_ 155.67 ± 26.55 ms, $Shank3_{30\text{ ppm}}^{\text{ex1}3-16-/-}$ 248.62 ± 38.67 ms, *p* < 0.05). Increasing dietary zinc has no effect on NMDAR EPSC decay tau in WT animals (Figure 5B; WT_150 ppm_ 150.04 ± 19.27 ms), however increased dietary zinc did reverse the decay tau in *Shank3*^ex13–16−/−^ mice such that the NMDAR decay kinetics were no longer significantly increased compared to WT mice, but were significant decreased in comparison to *Shank3*^ex13–16−/−^ mice fed normal zinc levels (Figures 5B,C; $Shank3_{150\text{ ppm}}^{\text{ex1}3-16-/-}$ 98.90 ± 10.25 ms, *p* < 0.0001).

**Figure 6:**
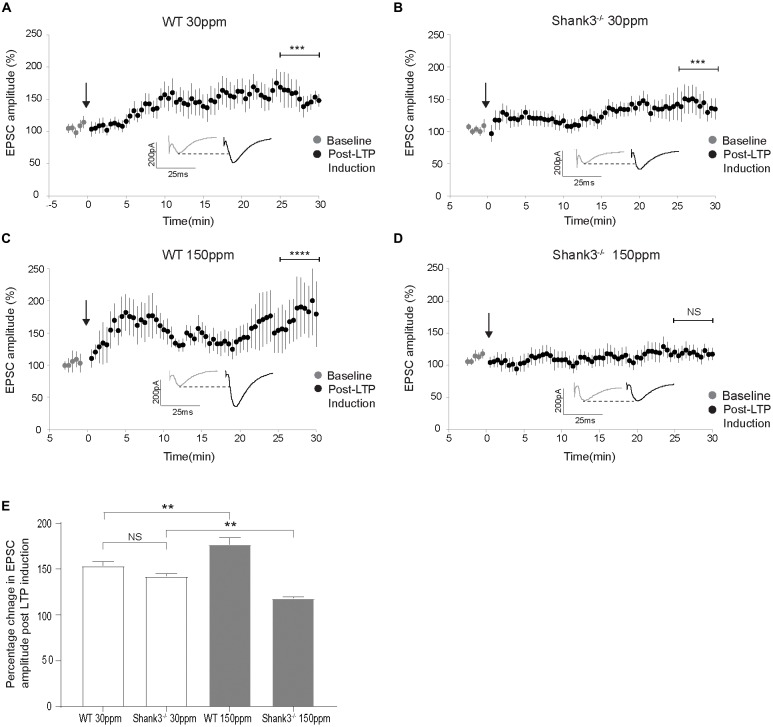
Dietary zinc supplementation prevents long term potentiation (LTP) in the cortico-striatal pathway in *Shank3*^ex13–16−/−^ mice. **(A–C)** WT_30 ppm_, WT_150 ppm_, and $Shank3_{30\text{ ppm}}^{\text{ex1}3-16-/-}$ mice all express LTP in response to tetanic stimulation (paired *t*-test, WT_30 ppm_ *n* = 11 cells from six animals, $Shank3_{30\text{ ppm}}^{\text{ex1}3-16-/-}$ *n* = 16 cells from six animals, WT_150 ppm_ *n* = 7 cells from four animals). **(D)** No significant change in AMPAR EPSC amplitude was observed 30 min after LTP induction in *Shank3*^ex13–16−/−^ mice with dietary zinc supplementation ($Shank3_{150\text{ ppm}}^{\text{ex1}3-16-/-}$: paired *t*-test, n =11 cells from five animals). **(E)** Bar graph of average percent change in AMPAR EPSC amplitude measured at 30 min post LTP-induction. All data represent mean ± SEM. **p < 0.01, ***p < 0.001, ****p < 0.0001, NS, not significant.

The cortico-striatal pathway is linked to compulsive grooming behaviors and habit learning (Lewis and Kim, 2009). Because we observed that repetitive grooming behaviors could be prevented by increased dietary zinc in *Shank3*^ex13–16−/−^ mice (Figure 1), and that dietary zinc significantly decreases NMDAR-mediated currents in *Shank3*^ex13–16−/−^ mice (Figure 5), we next examined whether dietary zinc also alters NMDAR-dependent synaptic plasticity in this pathway in WT and *Shank3*^ex13–16−/−^ mice fed with normal and supplemented zinc levels (Figures 6A–E). LTP was readily induced in the cortico-striatal pathway in WT mice fed 30 ppm zinc. Application of 4 × 100 Hz presynaptic stimulation trains for 1 s together with postsynaptic depolarization induced a significant increase in the AMPAR EPSC amplitude measured 30 min post stimulation in WT mice fed normal and increased zinc levels, and in *Shank3*^ex13–16−/−^ mice fed normal zinc levels (% baseline AMPAR EPSC amplitude: WT_30 ppm_ 153.57 ± 4.31%, *p* < 0.001; WT_150 ppm_ 176.93 ± 7.41%, *p* < 0.0001; $Shank3_{30\text{ ppm}}^{\text{ex1}3-16-/-}$ 142.20 ± 2.68%, *p* < 0.001; Figures 6A–C,E). In contrast, LTP was not able to be induced in the cortico-striatal pathway in *Shank3*^ex13–16−/−^ mice fed increased zinc levels, with no significant difference in AMPAR mediated EPSC amplitudes measured after the LTP induction protocol was applied (Figures 6D,E; Average AMPAR EPSC amplitude was 118.30 ± 1.19% of baseline at 30 min post-LTP induction; *p* > 0.05).

### Dietary Zinc Increases Synaptic Recruitment of Zinc-Responsive SHANK2

**Figure 7:**
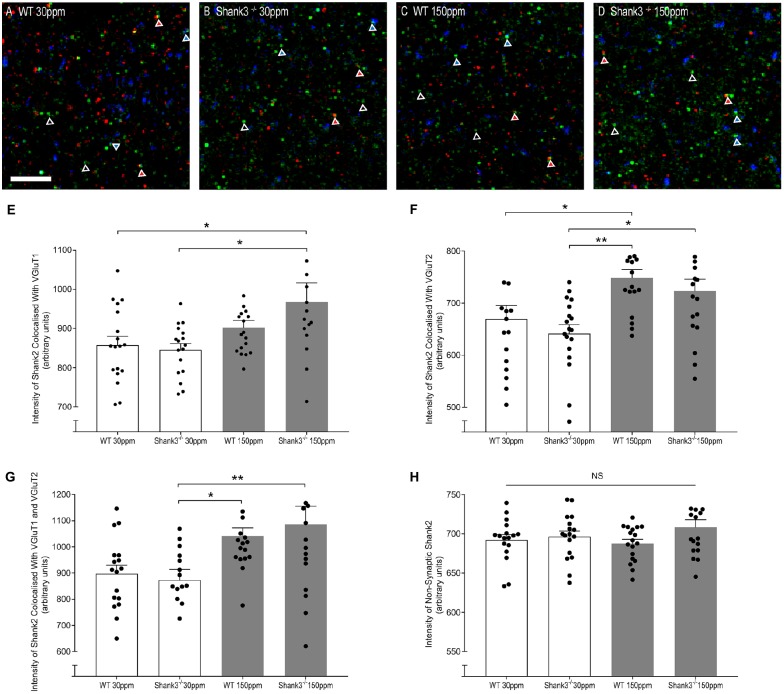
Dietary zinc supplementation increases cortico-striatal and thalamo-striatal synaptic SHANK2 expression in *Shank3*^ex13–16−/−^ mice. **(A–D)** Example overlaid images from WT and *Shank3*^ex13–16−/−^ mice fed normal (30 ppm) or high dietary zinc (150 ppm), immunostained for SHANK2 (green), VGluT1 (red) or VGluT2 (blue). Red triangles denote example synaptic SHANK2 puncta colocalized with VGluT1, blue triangles denote example synaptic SHANK2 puncta colocalized with VGluT2, open triangles denote example non-synaptic SHANK2 puncta. Scale bar 5 μm. **(E)** SHANK2 puncta intensity at cortico-striatal synapses, i.e., puncta co-localized with VGluT1, was significantly increased in $Shank3_{150\text{ ppm}}^{\text{ex168}3-16-/-}$ mice. **(F)** SHANK2 puncta intensity at thalamocostriatal synapses, i.e., puncta co-localized with VGluT2, was significantly increased in WT and *Shank3*^ex13–16−/−^ mice fed 150 ppm dietary zinc. **(G)** Total synaptic SHANK2, puncta co-localized with VGluT1 plus VGluT2, was significantly increased in both WT and *Shank3*^ex13–16−/−^mice fed 150 ppm dietary zinc. **(H)** Non-synaptic SHANK2, i.e., puncta not co-localized with either VGluT1 or VGluT2, was not significantly different between WT and *Shank3*^ex13–16−/−^ mice fed either diet. Data represent mean ± SEM from six *Shank3*^ex13–16−/−^ mice and six WT mice. Statistical significance was determined by one-way ANOVA with Tukey’s multiple comparisons test. *p < 0.05, **p < 0.01. NS, not significant.

The rescue of ASD-related behaviors in *Shank3*^ex13–16−/−^ mice suggests that other zinc-sensitive synaptic proteins may respond to increased dietary zinc. Like SHANK3, the SAM domain of SHANK2 also binds zinc (Boeckers et al., 2005). To explore the possibility that high dietary zinc increases the recruitment of zinc-sensitive SHANK2, we performed immunostaining to examine SHANK2 expression from WT and *Shank3*^ex13–16−/−^ mice fed with normal and supplemented zinc levels. Our data show that synaptic SHANK2 intensity levels are significantly higher in mice fed 150 ppm zinc (Figure 7). Synaptic SHANK2, defined as SHANK2 puncta that co-localized with the synaptic markers VGluT1 or VGluT2 (to identify cortico-striatal and thalamo-striatal synapses respectively; Fremeau et al., 2001), was observed to increase in intensity in *Shank3*^ex13–16−/−^ mice in response to dietary zinc supplementation (Figures 7A–H; At VGluT1 positive cortico-striatal synapses: WT_30 ppm_ 857.47 ± 22.41, $Shank3_{30\text{ ppm}}^{\text{ex1}3-16-/-}$ 845.57 ± 15.90; $Shank3_{150\text{ ppm}}^{\text{ex1}3-16-/-}$ 967.28 ± 48.76, *p* < 0.05, Figure 7E. At VGluT2 positive thalamo-striatal synapses: WT_30 ppm_ 669.53 ± 25.90, $Shank3_{30\text{ ppm}}^{\text{ex1}3-16-/-}$ 641.38 ± 17.08; $Shank3_{150\text{ ppm}}^{\text{ex1}3-16-/-}$ 722.99 ± 22.94, *p* < 0.05, Figure 7F). High dietary zinc also increased synaptic SHANK2 puncta intensity in WT mice, specifically at VGluT2 positive thalamo-striatal synapses (WT_150 ppm_ 748.87 ± 15.89, *p* < 0.05 compared with WT_30 ppm_) but not significantly at VGluT1 positive cortico-striatal synapses (WT_150 ppm_ 902.30 ± 18.17, *p* > 0.05 compared with WT_30 ppm_). SHANK2 increases were specific to synaptic sites, as no significant increases in SHANK2 were observed at non-synaptic sites (defined as SHANK2 puncta that did not colocalize with either VGluT1 or VGluT2; Figure 7); WT_30 ppm_ 692.31 ± 6.69, $Shank3_{30\text{ ppm}}^{\text{ex1}3-16-/-}$ 696.44 ± 7.17; WT_150 ppm_ 687.69 ± 5.31; $Shank3_{150\text{ ppm}}^{\text{ex1}3-16-/-}$ 708.33 ± 9.72; *p* > 0.05, Figure 7H).

## Discussion

Zinc is a known modulator of ion channels, synaptic transmission, and the recruitment of postsynaptic signaling protein complexes including SHANK2 and SHANK3 (Westbrook and Mayer, 1987; Chen et al., 1997; Yamada et al., 2002; Baron et al., 2006; Grabrucker et al., 2014; Arons et al., 2016). Interestingly, zinc responsiveness is maintained in SHANK3 containing ASD-associated mutations, and also in *Shank2*^−/−^ ASD mice (Lee et al., 2015; Arons et al., 2016), implying that zinc restores loss of function at synapses by enhancing the activity of SHANK3 and/or SHANK2. Here we show that increasing dietary zinc reverses synaptic and behavioral changes in the *Shank3*^ex13–16−/−^ model of ASD. Specifically, our data show that dietary zinc supplementation in *Shank3*^ex13–16−/−^ mice prevents ASD-related repetitive and anxiety behaviors as well as social deficits, in parallel with increasing the synaptic recruitment of zinc-sensitive SHANK2, decreasing NMDAR-EPSC amplitudes, and altering NMDAR decay kinetics. In addition, increased dietary zinc altered LTP at cortico-striatal synapses in *Shank3*^ex13–16−/−^ mice, a glutamatergic pathway implicated in repetitive behaviors (Lewis and Kim, 2009). Together these data suggest that increased dietary zinc affects synaptic and network function at synapses with reduced SHANK3 function by changing the functionality of synaptic zinc signaling systems. These appear to include alterations in NMDAR function that could contribute to the lack of cortico-striatal LTP (Calabresi et al., 1992) and thus ASD repetitive behaviors.

### Differential Effects of Changes in Dietary Zinc

In our studies, we observed that increases in dietary zinc also prevented anxiety behaviors and social novelty recognition deficits in *Shank3*^ex13–16−/−^ mice. Previous studies support the role of the amygdala in anxiety behaviors (Kalin et al., 2004; Etkin et al., 2009) as well as regulating social behaviors, especially those involving social novelty, with the most severe autistic-like social deficits occurring with combined damage to the amygdala and hippocampus (Sweeten et al., 2002). Dietary zinc supplementation therefore also likely alters glutamatergic synapses in the amygdala and/or hippocampus. Indeed, we have previously shown that zinc strengthens glutamatergic synaptic transmission in hippocampal neurons expressing SHANK3 ASD-associated mutations (Arons et al., 2016). Zinc mobilization has also been shown to restore glutamatergic synaptic transmission in the amygdala of *Tbr1*^−/−^ ASD mice and in the hippocampus of *Shank2*^−/−^ mice that was accompanied by a significant improvement in social interaction, but not anxiety behaviors, in both ASD mice models (Lee et al., 2015). The zinc responsiveness of the striatum, hippocampus, and amygdala together could underpin the normalization of ASD behaviors observed in these studies, although a complex balance likely exists between these key brain regions with respect to the zinc-induced changes at the synaptic, network and behavioral levels. The timing of zinc treatment also appears to influence the efficacy of the reversal of ASD-related anxiety behaviors, as certain ASD behaviors are irreversible in older mice (Mei et al., 2016). Therefore, a critical factor in the reversal of the observed social, anxiety, and repetitive ASD-related behaviors may be the increase in dietary zinc from weaning age.

### NMDARs Are a Major Target for Dietary Zinc Changes

While the observed deficits in AMPAR currents may underpin ASD-related behaviors in *Shank3*^ex13–16−/−^ mice, the dietary zinc reversal of ASD behaviors occurs concurrently with alterations specifically in NMDAR currents, suggesting that rescue does not involve zinc-dependent changes in AMPAR-mediated synaptic transmission. A significant difference in the dietary zinc-induced effects we describe here on the cortico-striatal pathway is the direction of change in synaptic strength, with a marked zinc-induced decrease in NMDAR currents and LTP occurring in this region. This is in contrast to treatment-induced increases in NMDAR mediated currents observed concurrently with reversal of ASD-related behaviors in other ASD mouse models (e.g., Won et al., 2012; Lee et al., 2015). With regards to zinc-specific treatments, these data show that zinc differentially alters synaptic function in the dorsolateral striatum compared with the hippocampus and amygdala where increases in NMDAR and AMPAR currents were observed (Lee et al., 2015; Arons et al., 2016). Even within the hippocampus zinc differentially alters LTP, promoting presynaptic mossy fiber LTP but inhibiting postsynaptic LTP (Pan et al., 2011). How the dietary zinc-induced decrease in NMDAR currents and lack of LTP in the cortico-striatal pathway contributes to changes ASD-associated behaviors is unknown. As this form of LTP is present in WT animals without ASD-related behaviors, we predict that the zinc-induced decrease in synaptic weight alters striatal outputs to the basal ganglia and motor circuitry via the thalamus to normalize ASD behaviors. While we present here an NMDAR-linked pathway for the preventative effect of dietary zinc on grooming behavior at cortico-striatal synapses, a deeper understanding of the role of zinc at specific synapses in multiple brain regions will be important to decipher the differential effects of zinc on specific ASD-related behaviors.

Our data also show that the significantly decreased NMDAR current amplitude induced by dietary zinc in *Shank3*^ex13–16−/−^ mice was accompanied by a lengthening of the decay time of NMDAR currents. This supports a mechanism whereby zinc induces a decrease in NMDAR synaptic expression and/or channel conductance, as well as a change in NMDAR subunit composition from GluN2B to GluN2A-containing receptors in *Shank3*^ex13–16−/−^ mice (Monyer et al., 1994; Vicini et al., 1998; Cull-Candy et al., 2001). As the dietary zinc effect was observed to specifically occur in *Shank3*^ex13–16−/−^ but not WT mice, the mechanisms underpinning changes induced by dietary zinc appear to require the absence of *Shank3* to exert their behavioral and synaptic effects. At WT synapses, SHANK3 has been shown to enhance the localization and stabilization of synaptic NMDAR expression by crosslinking NMDAR/PSD95 complexes with the underlying actin cytoskeleton (Naisbitt et al., 1999; Duffney et al., 2013). Synapses lacking SHANK3 will lack these cross-linkages, potentially leading to a decrease in the synaptic stability of NMDAR complexes. Additional zinc-sensitive signaling systems contributing to decreased NMDARs include a direct effect on the ion channels themselves, and/or zinc-dependent regulation of other zinc-dependent postsynaptic proteins such as SHANK2 or SAP102 (Firestein et al., 2000; Baron et al., 2006). With regards to the former, NMDARs contain zinc sensors in their N-terminal domains, enabling these receptors to detect zinc over a wide concentration range (Rachline et al., 2005). Zinc is a well-known inhibitor of NMDARs (Peters et al., 1987; Westbrook and Mayer, 1987; Mayer et al., 1989; Chen et al., 1997; Paoletti et al., 1997), particularly GluN2A-containing NMDARs which exhibit a higher zinc sensitivity (Chen et al., 1997; Rachline et al., 2005). This inhibition of NMDARs by zinc is decreased by PSD95 (Yamada et al., 2002). Therefore, the lack of SHANK3 crosslinked NMDAR/PSD95 complexes could also increase zinc inhibition of GluN2A-containing NMDARs, together resulting in decreased NMDAR-mediated currents and plasticity observed in *Shank3*^ex13–16−/−^ mutant mice fed high dietary zinc.

An important observation from our studies is that normalization of ASD-related behaviors can occur in the absence of *Shank3^ex13–16^*. However, this only became evident when dietary zinc levels were increased. These data suggest that there are still zinc sensitive molecules in *Shank3*^ex13–16−/−^ mice that can compensate at least in part for the loss of the SHANK3α and SHANK3β isoforms. A reduced level of the SHANK3γ isoform persists at the PSD in *Shank3*^ex13–16−/−^ mice (Peça et al., 2011), which may continue to respond to zinc-induced stabilization and recruitment of SHANK3 complexes (Baron et al., 2006; Arons et al., 2016). Moreover, an extensive array of SHANK3 isoforms has been described that are differentially regulated by activity, development, and epigenetics (Wang et al., 2014). Whether specific isoforms differentially contribute to zinc-responsiveness will also be of significant interest, especially splice isoforms within the zinc-binding SAM domains (Wang et al., 2014). SHANK2 may also compensate for the loss of *Shank3*^ex13–16−/−^ with dietary zinc, as SHANK2 binds zinc and shows zinc-dependent synaptic recruitment (Grabrucker et al., 2014). Higher levels of SHANK2 have also been described in *Shank3αβ*^−/−^ mice (Schmeisser et al., 2012), although this was not evident in our striatal immunohistochemical analysis in *Shank3*^ex13–16^ mice, similar to that described in *Shank3*^InsG3680+/+^ and *Shank3*^fx/fx^ mutant mice (Mei et al., 2016; Zhou et al., 2016). Specifically, our data show that increased dietary zinc levels drive the recruitment of SHANK2 to cortico-striatal and thalamo-striatal synapses. SHANK2 plays a major role in the early steps of PSD assembly, before PSD95 and NMDAR complexes form (Bresler et al., 2004), followed by later recruitment of SHANK3 driving synaptic formation and maintenance, then lastly SHANK1 driving synaptic maturation (Roussignol et al., 2005; Grabrucker et al., 2011a). The observed increase in SHANK2 with dietary zinc may facilitate early postsynapse formation and contribute to normalization of ASD behaviors, but it does not appear to replace the role of SHANK3 in crosslinking and stabilizing PSD95-NMDAR complexes. Zinc may also engage SHANK2 in other brain regions such as the hippocampus and amygdala to restore normal behaviors. Animal models lacking both *Shank2* and *Shank3* will therefore be of interest to examine whether loss of both SHANK proteins further impairs the zinc sensitivity of these behaviors. It will also be of importance to determine whether increases in dietary zinc can prevent ASD behavioral deficits in mouse models expressing other ASD mutations in SHANKs (e.g., Wang et al., 2011; Schmeisser et al., 2012; Won et al., 2012; Mei et al., 2016; Zhou et al., 2016) and also in other synaptic proteins (Ebert and Greenberg, 2013; Chen et al., 2014; Golden et al., 2018), to identify the breadth of dietary zinc as a potential therapeutic strategy, and the involvement of zinc-responsive SHANKs in restoring normal behaviors.

### Importance of Dietary Zinc Levels

The observation that dietary zinc levels can significantly alter rodent behavior is important not only in the context of reversing ASD behaviors, but these data also have wider significance when comparing behavioral phenotypes in mouse models fed different “control” diets. Dietary zinc levels vary significantly between control diets sold commercially: an online search of control rodent diet chow composition reveals that different companies provide standard chow with zinc levels that range between 25 ppm and 120 ppm. This >4-fold difference in zinc levels could significantly alter rodent behavioral phenotypes, adding variability in the reproducibility of disorder-specific phenotypes and efficiency of treatment strategies across different laboratories. Indeed, discrepancies in ASD phenotypes have been observed in identical ASD mouse models, including the *Shank3*^ex13–16−/−^ mutant mice employed in this study (Peca et al., 2001; Dhamne et al., 2017; Kabitzke et al., 2018). Recent studies have described these mice as having only “subtle” or “weak” social deficits (Dhamne et al., 2017; Kabitzke et al., 2018). We also observed limited social phenotypes in the *Shank3*^ex13–16−/−^ mutant mice, as both WT and *Shank3*^ex13–16−/−^ mutant mice spent significantly more time in close interaction with the stranger mouse compared to the empty chamber (Figure 3). However, we did observe a social novelty deficit in *Shank3*^ex13–16−/−^ mutant mice consistent with Peça et al. (2011) but in contrast to Kabitzke et al. (2018), that was prevented by increased dietary zinc. These differences, and their responsiveness to dietary zinc, further highlight the importance of identifying dietary zinc levels in normal chow across studies, as these levels could underpin differences in ASD phenotypes such as sociability deficits.

Zinc deficiency has been observed in humans affected not only by ASD, but also in Phelan-McDermid syndrome in which heterozygous loss of *SHANK3* occurs (Faber et al., 2009; Yasuda et al., 2011; Grabrucker et al., 2014; Pfaender et al., 2017). Interestingly, SHANK3 is expressed in the gut epithelium in mice and in human induced enterocytes (Pfaender et al., 2017), suggesting its reduction in PMS patients and in ASD mice with *Shank3αβ* deletions may contribute to altered gut function in these disorders. A parallel reduction in zinc transporters in SHANK3 complexes (*ZIP2* and *ZIP4*; Pfaender et al., 2017) may therefore contribute to zinc deficiencies in ASD and PMS. The behavioral and synaptic responsiveness of *Shank3*^ex13–16−/−^ mice to increased dietary zinc levels that were observed in the current study reflects an adequate level of gastrointestinal zinc absorption does occur in the gut of these mice. Therefore, despite a potential downregulation of specific zinc transporters, dietary zinc supplementation can overcome this deficit to influence ASD-related symptoms.

In summary, we have revealed that increased dietary zinc induces changes in synapse function and plasticity that occur in parallel with the reversal of ASD-related behaviors. Together these data identify the potential of chronically increasing dietary zinc as a viable strategy for altering cortico-striatal synaptic function and reversing specific behaviors related to ASD and Phelan-McDermid Syndrome.

## Author Contributions

CF performed experiments in Figures 1–6 and analyzed data. JM designed the study and wrote the manuscript. YV and KL performed experiments in Figure 7. YJ assisted with electrophysiology experiments in Figures 4–6. CG assisted with the design of the study and the writing of the manuscript.

## Conflict of Interest Statement

The authors declare that the research was conducted in the absence of any commercial or financial relationships that could be construed as a potential conflict of interest.
